# Integrated analysis identifies a novel lncRNA prognostic signature associated with aerobic glycolysis and hub pathways in breast cancer

**DOI:** 10.1002/cam4.4291

**Published:** 2021-09-27

**Authors:** Zheng Li, Juan Zheng, Yang Feng, Yaming Li, Yiran Liang, Ying Liu, Xiaolong Wang, Qifeng Yang

**Affiliations:** ^1^ Department of Breast Surgery, General Surgery, Qilu Hospital, Cheeloo College of Medicine Shandong University Jinan Shandong China; ^2^ Department of Ultrasound Qilu Children's Hospital of Shandong University Jinan Shandong China; ^3^ Pathology Tissue Bank Qilu Hospital of Shandong University Jinan Shandong China; ^4^ Research Institute of Breast Cancer Shandong University Jinan Shandong China

**Keywords:** bioinformatics, biomarkers, breast cancer, lncRNA, Prognosis

## Abstract

Long noncoding RNAs (lncRNAs) play a crucial role in cancer aerobic glycolysis. However, glycolysis‐related lncRNAs are still underexplored in breast cancer. In this study, we identified the five most glycolysis‐related lncRNAs in breast cancer to construct a prognostic signature, which could distinguish between patients with unfavorable and favorable prognoses. To investigate the role of signature lncRNAs in breast cancer, we profiled their expression levels in breast cancer progression cell line model. Real‐time PCR revealed that the five lncRNAs could contribute to breast cancer initiation or progression. Furthermore, we observed that the levels of four lncRNAs expression had a significant trend of gradient upregulation with the addition of glycolysis inhibitor in breast cancer cells. Afterward, random forest and logistic regression were conducted to assess the model's performance in stratifying glycolysis status. Finally, a nomogram including the lncRNA signature and clinical features was developed, and its efficacy in predicting the survival time and clinical utility was evaluated using a calibration curve, concordance index, and decision curve analysis. In this study, gene set enrichment analysis showed that the mTOR pathway, a central pathway in tumor initiation and progression, was significantly enriched in the high‐risk group. In addition, gene set variation analysis was performed to validate our findings in two independent datasets. Subsequent weighted gene co‐expression network analysis, followed by enrichment analysis, indicated that downstream cell growth‐related signaling was strikingly activated in the high‐risk group, and may directly promote tumor progression and escalate mortality risk in patients with high‐risk scores. Overall, our findings may provide novel insight into lncRNA‐related metabolic regulation, and help to develop promising prognostic indicators and therapeutic targets for breast cancer patients.

## Graphical abstract

In conclusion, we identified five glycolysis‐related lncRNAs to construct a lncRNA signature on the basis of the glycolysis score, which could predict survival probability and glycolysis status. Moreover, hallmark gene sets associated with cell growth were significantly activated in the high‐risk breast cancer patient subgroup. Overall, the lncRNA signature could function as a robust prognostic indicator and help develop novel therapeutic strategies for breast cancer patients.

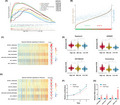

## 1 INTRODUCTION

Breast cancer is the most common cancer and the leading cause of cancer‐related death in women worldwide.^1^ Although conventional treatment strategies have been well applied, many patients with breast cancer still have unfavorable prognosis.^2^ Consequently, it is essential to further investigate novel prognostic indicators, diagnostic biomarkers, and therapeutic targets for improved clinical outcomes.

Altered energy metabolism is one of the pivotal fingerprints associated with cancer biological behaviors.^3^ Aerobic glycolysis, known as the ‘Warburg effect’, is a preferential metabolic phenotype for cancer cells.^4^ Although aerobic glycolysis has poor ATP production compared to mitochondrial oxidative phosphorylation, cancer cells accelerate the ATP production rate and increase glucose uptake via metabolic reprogramming.^5^ Meanwhile, glycolysis intermediates not only contribute to macromolecule formation in various biosynthetic pathways,^6^ but also induce resistance to chemotherapy and radiotherapy.^7^, ^8^ In addition, glycolysis provides a favorable tumor microenvironment for cancer cells to thrive.^9^ Due to the crucial role of tumor aerobic glycolysis in breast cancer initiation and progression, further exploration could help to improve clinical outcomes for patients with breast cancer.

To date, long noncoding RNAs (lncRNAs), which are RNAs longer than 200 nucleotides, have been shown to play an important role in transcription, post‐transcription, and epigenetic modification, and influence genes associated with glucose metabolism in several cancer types.^10^, ^11^, ^12^, ^13^, ^14^ In addition, lncRNAs could contribute to metabolism reprogramming, which could regulate carcinogenesis and progression by providing adequate nutrition for cancer cells to circumvent energy stress.^15^ Malakar et al. reported that lncRNA *MALAT1* may induce glucose metabolism reprogramming to promote tumor malignant progression by upregulating SRSF1 and activating the mTORC1‐4EBP1 axis in hepatocellular carcinoma.^16^ Li et al. demonstrated that lncRNA *UCA1* plays a positive role in glycolysis by upregulating hexokinase two through the mTOR‐STAT3/microRNA143 pathway in bladder cancer.^17^ Liu et al. revealed that downregulation of lncRNA *NBR2* could attenuate AMPK activation and promote mTORC1‐mediated protein synthesis and cancer cell growth under glucose‐starved stress.^18^ Additionally, Hung et al. suggested that lncRNA *PCGEM1* may function as a crucial transcription regulator in central metabolic pathways, and promote cancer cell proliferation by regulating tumor metabolism via co‐activation of both c‐Myc and androgen receptor (AR).^19^ Hence, glycolysis‐related lncRNAs could provide novel insights for further exploration of metabolic strategies in breast cancer prognosis and treatment.

In this study, we applied integrated bioinformatics analysis to identify a prognostic signature of five glycolysis‐related lncRNAs, which could predict the survival time and glycolysis status in breast cancer patients. Moreover, we further investigated the potential biological roles underlying the lncRNA signature via systematic bioinformatics analysis and in vitro experiments. Thus, our findings provide a novel insight into lncRNA‐related metabolic regulation and help to develop promising prognostic indicators and therapeutic targets for breast cancer patients.

## 2 MATERIALS AND METHODS

### 2.1 Sample datasets and data processing

The Cancer Genome Atlas (TCGA) data portal (https://portal.gdc.cancer.gov/) was used to obtain TCGA RNA‐Seq dataset. The raw count data were transformed through the variance‐stabilizing transformation method using the DESeq2^20^ package and then were quantile‐normalized using the preprocessCore package. The 888 cases of breast cancer obtained from the TCGA were screened based on the following inclusion criteria: availability of complete data on overall survival time, survival status, age, subtype, and AJCC stage. The molecular subtypes were classified by the PAM50 subtype predictor, including luminal A, luminal B, HER2‐enriched, basal‐like, and normal‐like. The METABRIC dataset with normalized data sourced from Molecular Taxonomy of Breast Cancer International Consortium (https://www.mbcproject.org/) contained 1903 breast cancer cases with overall survival time and survival status. The GSE20685 dataset was downloaded from the Gene Expression Omnibus (https://www.ncbi.nlm.nih.gov/gds/), which contained 327 breast cancer cases with overall survival time, survival status, age, and clinical stage. As previously described,^21^ SeqMap was used to reannotate the probe sets of the Affymetrix Hg‐U133 Plus 2.0 array. The microarray data were background corrected and normalized via the Limma package.^22^ The log2‐transformed normalized data were used for downstream analysis. In this study, TCGA and METABRIC datasets were used to assess the efficacy of the glycolysis score. The TCGA dataset served as the training set to select the five most glycolysis‐related lncRNAs to construct a prognostic signature. The GSE20685 dataset served as the validation set to validate our findings from the training set. The clinical information for the included patients is summarized in Table S1.

### 2.2 Cell lines and cell culture

All cell lines used in this study were purchased from American Type Culture Collection (ATCC), and maintained in a humidified incubator at 37℃ with 5% CO_2_. MDA‐MB‐231 and MDA‐MB‐468 cells were cultured in Dulbecco's modified Eagle's medium (DMEM) (Invitrogen), supplemented with 10% fetal bovine serum (Hyclone), 100 U/ml of penicillin, and 100 μg/ml of streptomycin. MCF‐10A, MCF‐10AT, and MCF‐10CA1A cells were cultured in DMEM/F12 (Invitrogen, Carlsbad, California) with 5% horse serum (Invitrogen), 500 ng/ml of hydrocortisone (Sigma‐Aldrich), 100 ng/ml of cholera toxin (Sigma‐Aldrich), 10 μg/ml of insulin (Invitrogen), and 20 ng/ml of epidermal growth factor (EGF, Sigma‐Aldrich).

### 2.3 RNA extraction, reverse transcription, and real‐time PCR analysis

Total RNA was extracted from breast cancer cells using the TRIzol reagent (Invitrogen). After RNA was reversely transcribed into complementary DNA (cDNA) by PrimeScript reverse transcriptase (RT) reagent kit (TaKaRa), Biosystems StepOne plus System was used to perform real‐time PCR assay. Primers used for real‐time PCR are listed in Table S2.

### 2.4 Lactate assay

The lactate level of cell supernatant was measured by the Lactate Assay Kit (Eton Bioscience). Briefly, breast cancer cells were seeded at a density of 20,000 cells per well in a 96‐well plate. The next day, the cell supernatant from each well was collected, mixed with l‐Lactate assay solution, and then incubated at 37℃ for 30 min. Lastly, the absorbance at 490 nm was read to measure the concentration of l‐Lactate.

### 2.5 3‐(4,5‐dimethylthiazol‐2‐yl)‐2,5‐diphenyl tetrazolium bromide (MTT) assay

Cell growth was measured using MTT assay. Briefly, breast cancer cells were seeded in 96‐well plates at a density of 2 × 10^3^ cells per well, treated with vehicle or 10 nM rapamycin for the indicated time, and then incubated with 20 μl MTT (5 mg/ml) for additional 4–6 h at 37℃ with 5% CO_2_. MTT reagent was aspirated and 100 μl of dimethyl sulfoxide (DMSO) was add into each well. Absorbance values were measured at 490 nm on an microplate reader (Bio‐Rad).

### 2.6 Development and evaluation of glycolysis score

First, glycolysis‐related genes were obtained from the Molecular Signatures Database (MSigDB) containing REACTOME_GLYCOLYSIS, HALLMARK_GLYCOLYSIS, and KEGG_GLYCOLYSIS_GLUCONEOGENESIS.^23^ Next, we calculated the glycolysis scores for each patient via single sample Gene Set Enrichment Analysis (ssGSEA). Based on the median glycolysis score, breast cancer patients were classified into two subgroups, high‐ and low‐glycolysis groups. Lastly, GSEA and Kaplan–Meier survival analysis were used to evaluate the efficacy of glycolysis scores in two independent datasets.

### 2.7 Construction of the lncRNA signature

Based on the criteria (|*r*| > 0.35, *p* value <0.001), a cohort of lncRNAs significantly associated with the glycolysis score was selected in the training set by Spearman's correlation analysis. Subsequently, univariate followed by stepwise multivariate Cox regressions were performed to identify the five most promising lncRNAs and develop a prognostic signature.

### 2.8 Prognostic lncRNA signature‐based risk score

Multivariate Cox model analysis using bidirectional stepwise selection was performed to calculate the regression coefficients for determining each lncRNA expression level. The risk score formula is: Risk score = (−0.249 × expression of AC007686.3) + (−0.253 × expression of BAIAP2‐DT) + (−0.210 × expression of LINC00926) + (0.056 × expression of LINC01016) + (−0.107 × expression of MAPT‐AS1).

### 2.9 Construction and evaluation of a nomogram

The RMS package was used to generate a nomogram, in which predictive accuracy and discrimination ability were evaluated by calibration curve and concordance index (C‐index), respectively. Moreover, a decision curve analysis (DCA) was performed to evaluate the clinical utility of the nomogram by quantifying the net benefits against a range of threshold probabilities.^24^

### 2.10 Gene set enrichment analysis and gene set variation analysis

Gene set enrichment analysis was performed by the JAVA program using the Hallmark gene sets sourced from MSigDB. All genes were ranked based on differential significance between the high‐ and low‐risk subgroups stratified by the median risk score. After performing 1000 permutations, gene set enrichment with nominal *p *< 0.05 and FDR < 0.25 was considered as a significant difference. In GSVA,^25^ Spearman's correlation analysis was carried out to assess the relationship between the risk score and specific hallmark gene sets in the training and validation sets.

### 2.11 Weighted gene correlation network analysis

Weighted Gene Correlation Network Analysis procedure was carried out as described previously.^26^ Briefly, a soft thresholding power of six was selected to generate a scale‐free topology from adjacency matrix. DeepSplit of 2 and minModuleSize of 30 were set as the parameters of the Dynamic Tree Cut method to avoid generating too many modules. The height cut‐off value was set to 0.25 to merge modules with similarity >0.75. Finally, the enrolled genes generated 17 modules (except the gray module) by cluster analysis. We evaluated the association between the risk score and module eigengenes (MEs) to achieve the module most closely related to the risk score. The hub genes were selected according to module membership (MM) greater than 0.8 and gene significance (GS) greater than 0.4. Biological process enrichment analysis of hub genes from highly related modules was performed using Metascape (http://metascape.org/).

### 2.12 Statistical analysis

Multivariate survival analysis for the lncRNA signature and clinicopathological features was performed using Cox proportional hazards regression models to determine which factors could act as an independent prognostic indicator. A time‐dependent receiver operating characteristic (ROC) analysis was conducted to investigate the model's predictive performance at 1, 3, 5, and 10 years. The Kaplan–Meier method combined with the log‐rank test was carried out to assess the overall survival time between the two subgroups. Two well‐established machine learning algorithms (random forest [RF] and logistic regression [LR]) were used to confirm the efficacy of the lncRNA signature for stratifying glycolysis status on the basis of the area under the ROC curve (AUC) score through fivefold cross validations.^27^ Logistic regression analysis was performed to evaluate whether the lncRNA signature had a better performance for stratifying the glycolysis status than individual lncRNA. Spearman's correlation analysis was used to assess the relationship among risk scores, glycolysis scores, and each lncRNA. The Chi‐squared test was used to examine the association between the lncRNA signature and clinicopathological phenotype based on the median risk score as a cutoff threshold. For continuous data, difference between two groups was assessed using the Student's *t*‐test or Wilcoxon test, and multiple groups comparison was made using the Kruskal–Wallis test. Experimental data are presented as mean (±SD). In this study, we used the R project (version 3.6.1) and GraphPad Prism 8 to perform the main statistical analysis. Differences with *p* < 0.05 were considered statistically significant.

## 3 RESULTS

### 3.1 Development and evaluation of the glycolysis score

**FIGURE 1:**
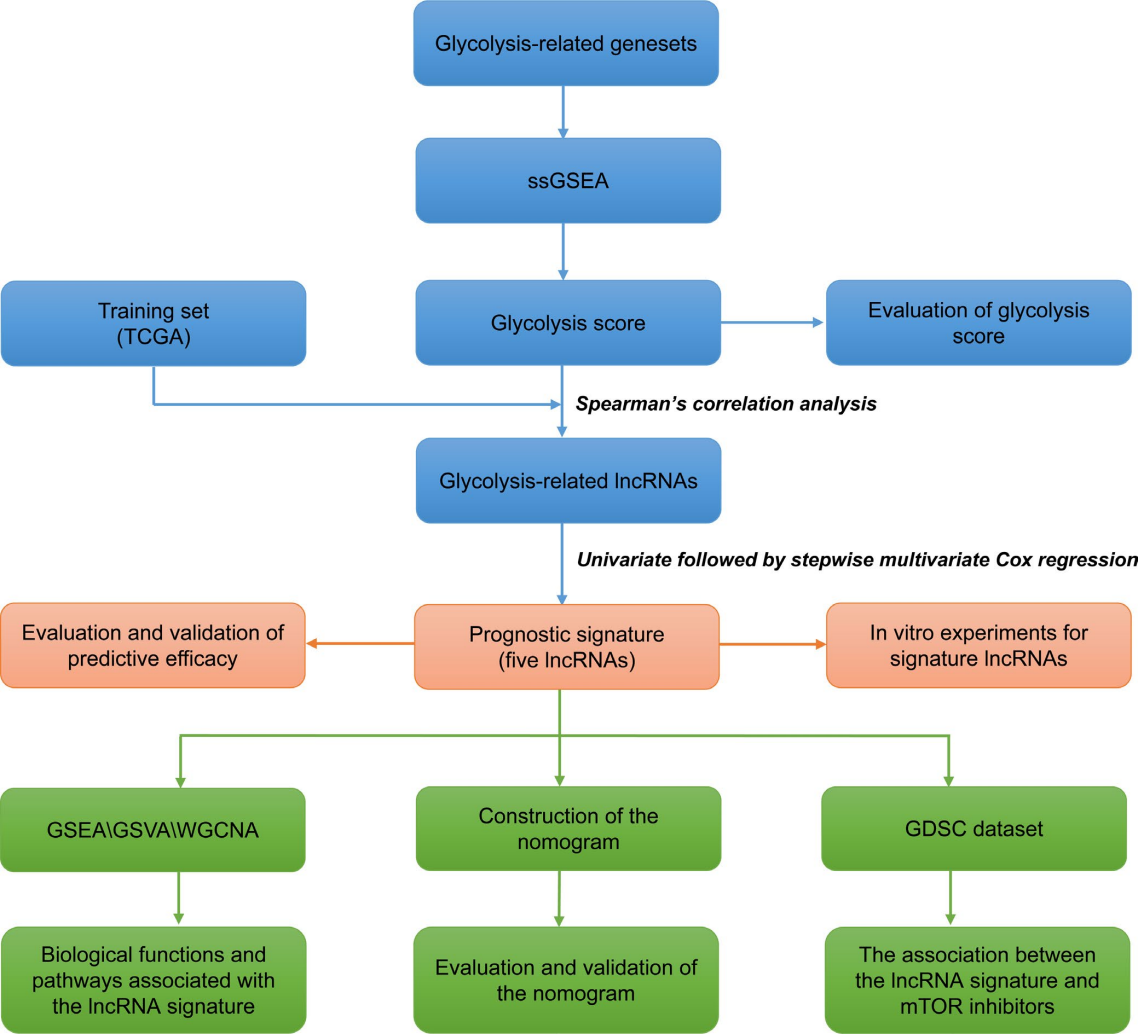
The flow diagram of our analysis procedure

**FIGURE 2:**
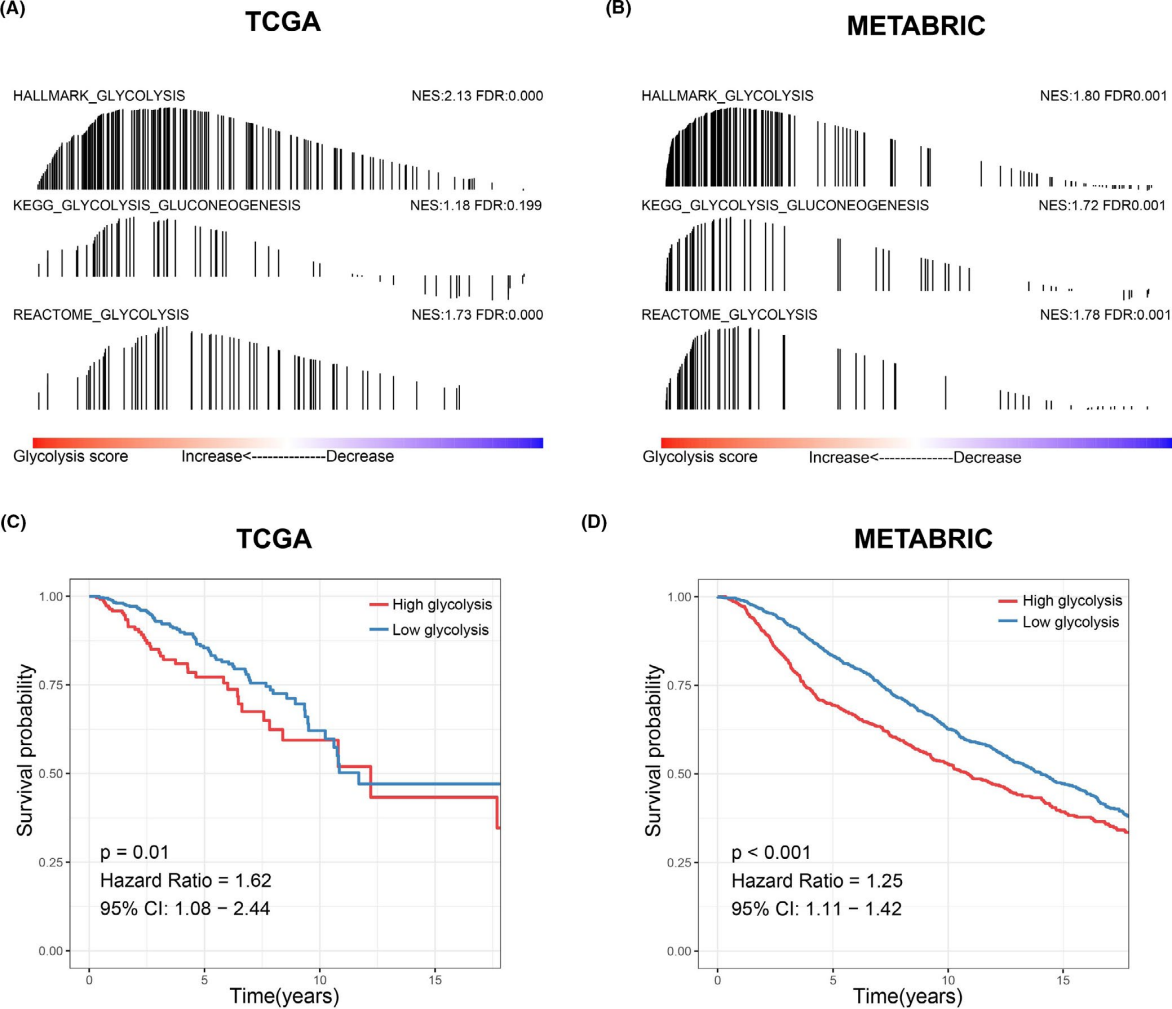
Development and evaluation of the glycolysis score. (A, B) Gene set enrichment analysis on the basis of glycolysis score. (C, D) Kaplan–Meier survival analysis on the basis of glycolysis score

A flow diagram illustrating our analysis procedure is shown in Figure 1. Here, TCGA and METABRIC datasets were employed to assess the efficacy of the glycolysis score (ssGSEA score). GSEA identified that three glycolysis‐related gene sets were significantly enriched in the high‐glycolysis group, which indicated that the glycolysis score could directly represent glycolysis status (Figure 2A,B). In addition, survival analysis revealed that patients with high‐glycolysis scores had shorter survival times than those with low‐glycolysis scores (Figure 2C,D).

### 3.2 Construction of a five glycolysis‐related lncRNA signature in the training set

**FIGURE 3:**
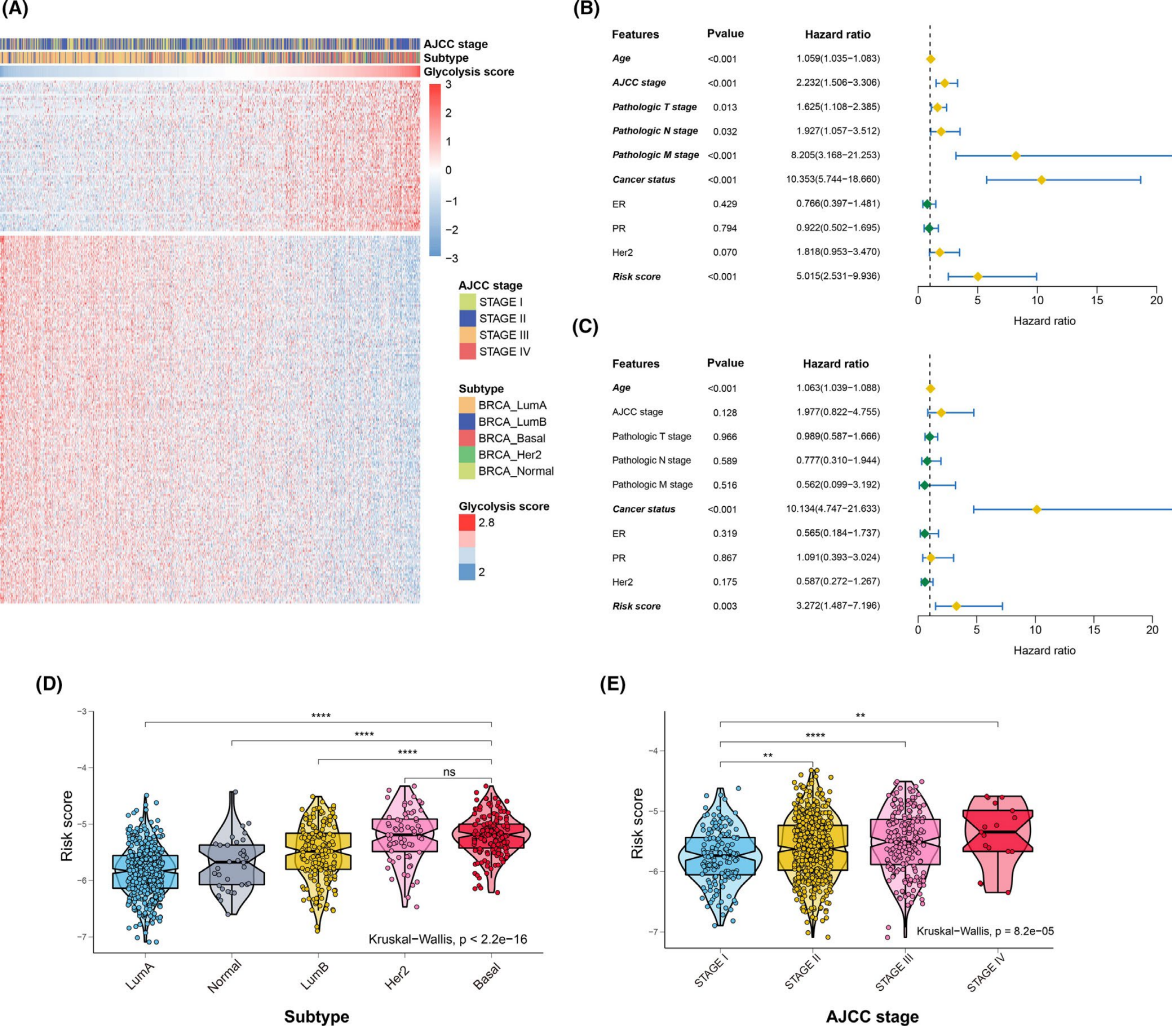
Construction of a five glycolysis‐related lncRNA signature in the training set. (A) A cohort of glycolysis‐related lncRNAs was identified by Spearman's correlation analysis. (B) Univariate Cox regression analysis. (C) Multivariate Cox regression analysis. Bold italics indicate statistically significant variables. (D) Comparison of risk scores associated with different clinical stages. (E) Comparison of risk scores associated with different molecular subtypes (*p < 0.05, **p < 0.01, ***p < 0.001, **** *p* < 0.0001; Wilcoxon test)

Based on the criteria (|*r*| > 0.35, *p* value <0.001), the 121 most glycolysis‐related lncRNAs were obtained from the training set using Spearman's correlation analysis (Figure 3A; Table S3). Univariate followed by stepwise multivariate Cox regression analyses were performed, and then a five glycolysis‐related lncRNA signature was constructed (Table S4). According to univariate Cox regression analysis, AC007686.3, BAIAP2‐DT, LINC00926, LINC01016, and MAPT‐AS1 were defined as protective factors (HR value <1) in the prognostic model (Table 1). As shown in Figure 3B, univariate Cox regression analysis was used to examine the effect of clinicopathologic features and lncRNA signature on overall survival in the TCGA cohort. Subsequent multivariate Cox regression analysis indicated that age, cancer status, and lncRNA signature had a significant association with duration of patient survival independent of other variables (Figure 3C). In addition, we found that the malignant grade of the AJCC stage was evidently associated with a high‐risk value (Figure 3D). Meanwhile, the molecular subtype of breast cancer was strikingly related to the risk score, which showed that basal‐like or HER2 patients had higher risk values than patients with other subtypes (Figure 3E).

**TABLE 1:** Top five glycolysis‐related lncRNAs identified from Spearman's correlation analysis and Cox regression analysis

lncRNA_symbol	Ensemble_ID	Spearman's correlation analysis		Univariate cox regression analysis			
		R	*p* value	HR	HR.95L	HR.95H	*p* value
AC007686.3	ENSG00000273729	−0.384	1.43E‐32	0.660	0.499	0.872	0.0034
BAIAP2‐DT	ENSG00000226137	−0.375	5.35E‐31	0.709	0.562	0.893	0.0035
LINC00926	ENSG00000247982	−0.403	4.69E‐36	0.740	0.625	0.876	0.0005
LINC01016	ENSG00000249346	−0.420	3.64E‐39	0.949	0.900	1.000	0.0492
MAPT‐AS1	ENSG00000264589	−0.364	3.69E‐29	0.889	0.834	0.947	0.0003

### 3.3 Investigation of the association between signature lncRNAs and glycolysis

**FIGURE 4:**
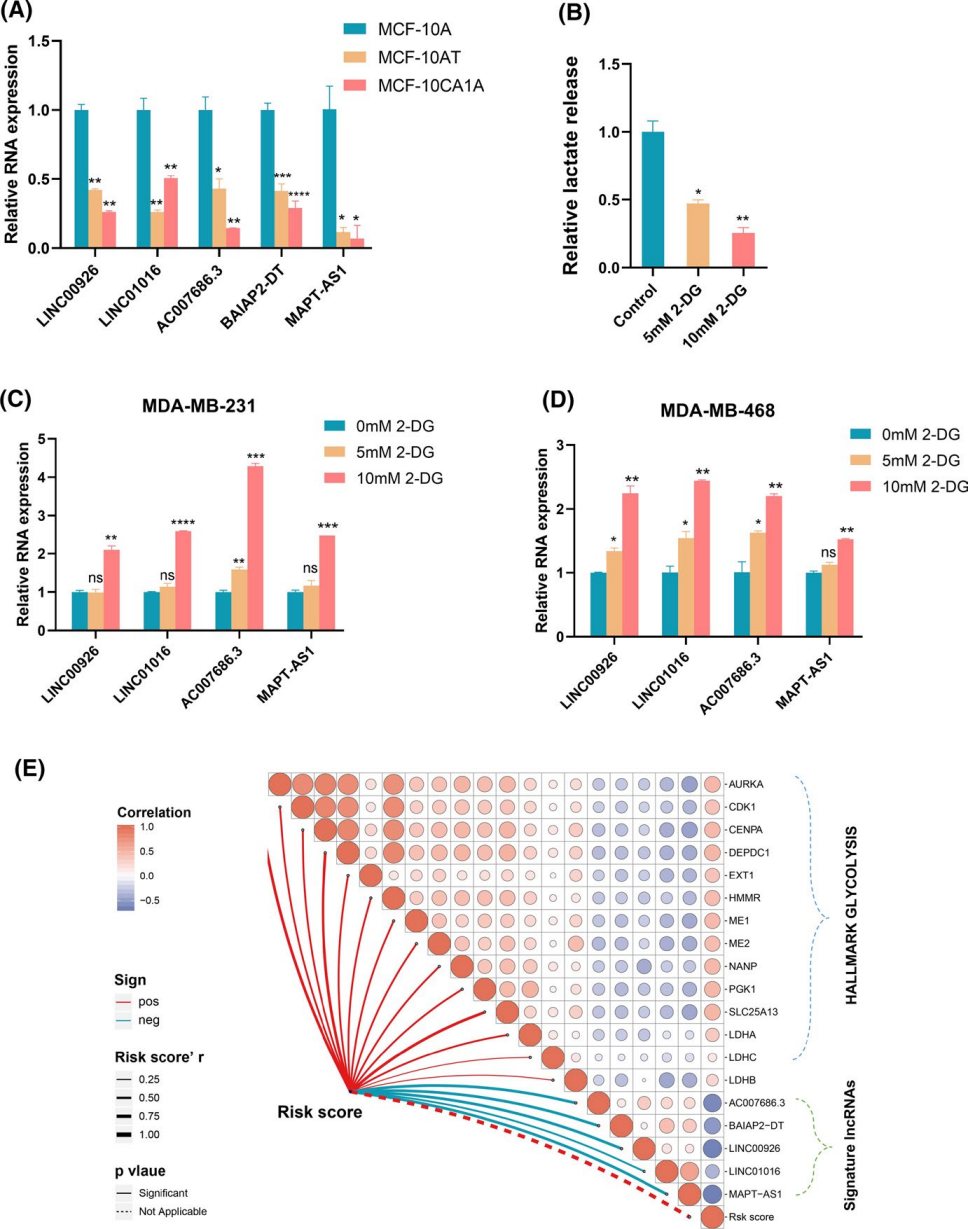
Investigation of the association between signature lncRNAs and glycolysis. (A) Expression profiles of signature lncRNAs in breast cancer progression cell line model. (B) The effect of 2DG on lactate generation was estimated in MDA‐MB‐468 cells. (C, D) Breast cancer cells treated with 2DG‐containing medium were subjected to real‐time PCR analysis to measure signature lncRNAs expression. (E) The relationship among aerobic glycolysis‐related factors, lncRNA signature, and each lncRNA (*p < 0.05, **p < 0.01, ***p < 0.001, ****p < 0.0001; Student's *t*‐test)

First, we evaluated the role of signature lncRNAs in breast cancer progression via breast cancer progression cell line model (MCF10A\MCF10AT\MCF10CA1A). Real‐time PCR suggested that the levels of five lncRNAs expression were reduced in premalignant MCF10AT and malignant MCF10CA1A cells compared to parental MCF10A cells (Figure 4A). Notably, LINC00926, AC007686.3, and BAIAP2‐DT appeared to be a significant trend of gradient downregulation from MCF10A to MCF10AT and MCF10CA1A cells, indicating that these three lncRNAs could play an important role in breast cancer initiation and progression. To further investigate the association of signature lncRNAs with glycolysis, we treated breast cancer cells with 2‐Deoxy‐D‐glucose (2DG), a glycolysis inhibitor. Lactate production was used to examine for levels of the glycolytic in breast cancer cells treated with 5 or 10 mM 2DG for 12–24 h. As presented in Figure 4B, gradient descent lactate production was observed with an increase in 2DG concentration. On the contrary, the use of 2DG enhanced expression levels of LINC00926, LINC01016, AC007686.3, and MAPT‐AS1 in MDA‐MB‐231 and MDA‐MB‐468 cells (Figure 4C,D). However, we did not observe significant changes in BAIAP2‐DT expression levels (data not shown). Afterward, we conducted Spearman's correlation analysis to further identify the relationship between aerobic glycolysis‐related factors and lncRNA signature in the training set. Our results showed that the risk score was positively correlated with hub glycolysis‐related genes, and signature lncRNAs had a negative correlation with them (Figure 4E). We next examined these correlations in the validation set (Figure S1A). A similar result was yielded to further support our findings from the training set.

### 3.4 Validation and further evaluation of the lncRNA signature in the training and validation sets

**FIGURE 5:**
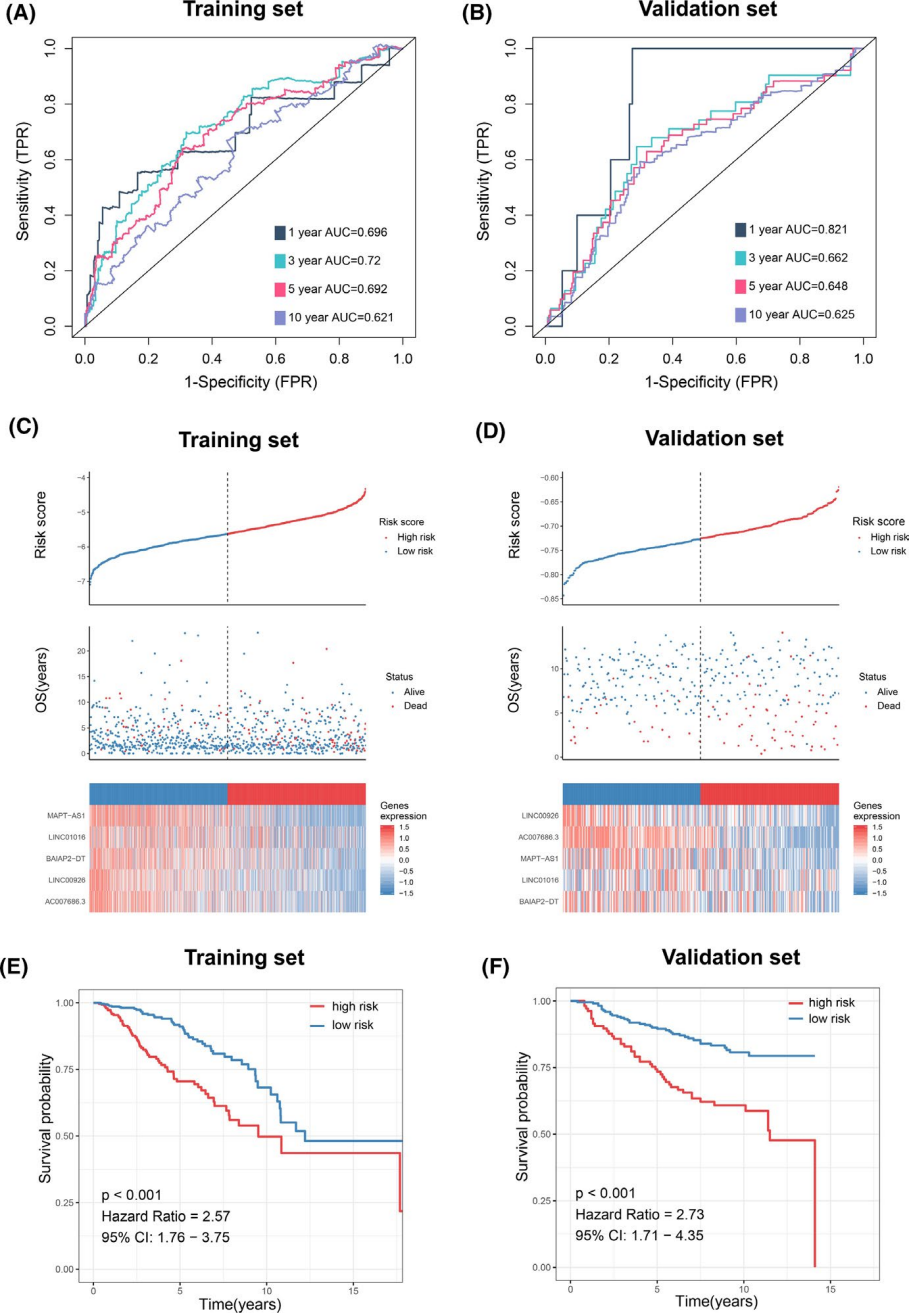
Validation of the lncRNA signature in the training and validation sets. (A, B) Time‐dependent ROC curves at 1, 3, 5, and 10 years. (C, D) From top to bottom are the risk scores, patients’ survival status distribution, and the expression heatmap of five glycolysis‐related lncRNAs in the low‐ and high‐risk groups. (E, F) Kaplan–Meier survival analysis on the basis of risk score

Here, ROC analysis was applied to assess the predictive accuracy of signature at 1, 3, 5, and 10 years. The area under the curve (AUC) scores in the training and validation sets are shown in Figure 5A,B, respectively. Using the median risk score as the cutoff threshold, the distribution of survival status, overall survival time, and lncRNA expression in the training and validation sets is presented separately in Figure 5C,D. Survival analysis showed that patients with high‐risk scores had poor survival time compared to those with low‐risk scores (Figure 5E,F).

**FIGURE 6:**
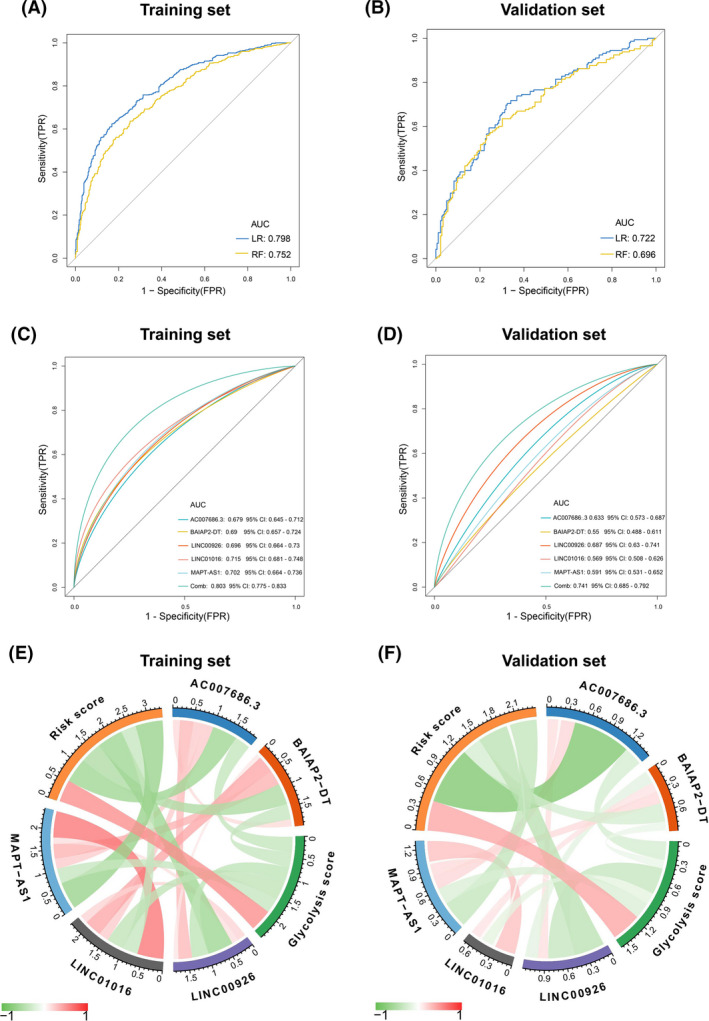
Further evaluation of the lncRNA signature in the training and validation sets. (A, B) Logistic regression (LR) and random forest (RF) were used to evaluate the signature's performance in stratifying glycolysis status. (C, D) Logistic regression analysis was used to identify that the lncRNA signature predicted glycolysis status more efficiently than individual lncRNA. (E, F) The interplay among risk scores, glycolysis scores, and five lncRNAs

Subsequently, the signature's predictive capability for glycolysis status was further assessed by RF and LR analyses. Importantly, moderate predictive performance for glycolysis status was observed in the training and validation sets (Figure 6A,B). Additionally, LR analysis identified that the lncRNA signature predicted glycolysis status more efficiently than individual lncRNA (Figure 6C,D). The interplay among risk scores, glycolysis scores, and five lncRNAs was further confirmed by Spearman's correlation analysis. It revealed that the risk score was positively associated with the glycolysis score, whereas five lncRNAs were negatively associated with the risk and glycolysis scores (Figure 6E,F). Additionally, the interactions among the five lncRNAs are shown in Figure 6E,F. Of note, the validation set had an acceptable performance with the training set.

### 3.5 Construction and evaluation of the nomogram

**FIGURE 7:**
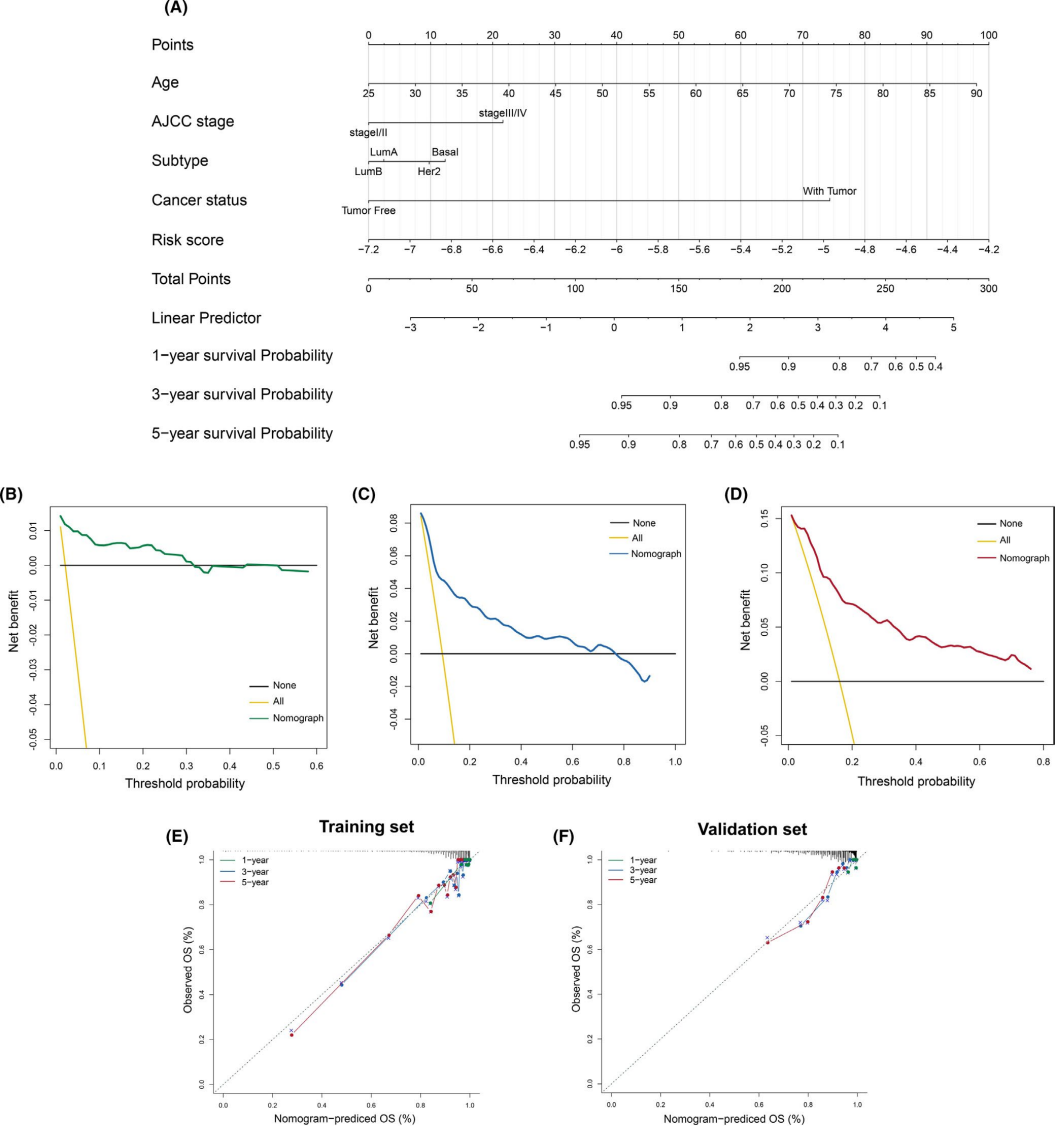
Construction and evaluation of the nomogram. (A) Construction of the nomogram. (B–D) Decision curve analysis associated with the nomogram at 1, 3, and 5 years. Note that yellow line: net benefit of all‐treat scheme; black line: net benefit of none‐treat scheme; green line: net benefit of the nomogram for predicting 1‐year survival time; blue line: net benefit of the nomogram for predicting 3‐year survival time; red line: net benefit of the nomogram for predicting 5‐year survival time. E‐F, Calibration curve of the nomogram in the training and validation sets. The prognostic model's performance at 1, 3, and 5 years is presented by green, blue, and red lines, respectively. And the gray dashed line of 45◦ represents the ideal performance

Before the signature was sent to construct a nomogram, we employed the Chi‐squared test to explore the association between the lncRNA signature and clinicopathological features by stratifying TCGA‐derived patients into high‐ and low‐risk groups based on the median risk score as a cutoff threshold. As shown in Table 2, the risk score was significantly associated with AJCC stage, cancer status, and subtype. To enhance the predictive efficacy of the lncRNA signature, we developed a nomogram that incorporated age, AJCC stage, subtype, cancer status, and the lncRNA signature (Figure 7A). DCA was performed to estimate net benefit and clinical utility for this nomogram at 1, 3, and 5 years (Figure 7B,D). It revealed that the nomogram displayed consistent positive and larger net benefit across a broad range of threshold probabilities (more than 70%) at 3 and 5 years compared to either the none‐treat scheme or all‐treat scheme. However, the 1‐year DCA showed that patients could only acquire net benefits within nearly 30% of the threshold probability. The calibration curve indicated that the nomogram survival prediction for breast cancer patients had an excellent agreement with actual observations at 1, 3, and 5 years, with a C‐index of 0.855 (95% CI, 0.812–0.898) (Figure 7E). Importantly, a good performance for predicting survival was also observed in the validation set, with a moderate discrimination (C‐index of 0.725 [95% CI, 0.671–0.779]) (Figure 7F).

**TABLE 2:** The chi‐squared test of the association between the lncRNA signature and clinicopathological features in TCGA breast cancer dataset

Features	Alive (768)	Dead with high risk (79)	Dead with low risk (41)	Total (888)	*p* value
Age					
Mean (SD)	57.5 (12.6)	60.2(15.4)	63.2(14.3)	58(13)	
Median (Min, Max)	57.5 (26, 89)	60 (26, 90)	66 (34, 88)	58 (26,90)	
Subtype					
BRCA_Basal	135 (17.6\%)	19 (24.1\%)	2 (4.9\%)	156 (17.6\%)	
BRCA_Her2	53 (6.9\%)	14 (17.7\%)	NA	67 (7.5\%)	
BRCA_LumA	406 (52.9\%)	22 (27.8\%)	29 (70.7\%)	457 (51.5\%)	
BRCA_LumB	146 (19.0\%)	20 (25.3\%)	8 (19.5\%)	174 (19.6\%)	
BRCA_Normal	28 (3.6\%)	4 (5.1\%)	2 (4.9\%)	34 (3.8\%)	0.004*
AJCC stage					
Stage I	142 (18.5\%)	8 (10.1\%)	7 (17.1\%)	157 (17.7\%)	
Stage II	459 (59.8\%)	36 (45.6\%)	17 (41.5\%)	512 (57.7\%)	
Stage III	163 (21.2\%)	26 (32.9\%)	13 (31.7\%)	202 (22.7\%)	
Stage IV	4 (0.5\%)	9 (11.4\%)	4 (9.8\%)	17 (1.9\%)	2.4e‐13*
Cancer status					
Tumor free	668 (87.0\%)	32 (40.5\%)	17 (41.5\%)	717 (80.7\%)	
With tumor	20 (2.6\%)	34 (43.0\%)	15 (36.6\%)	69 (7.8\%)	1.9e‐52*
Glycolysis status					
High	375 (48.8\%)	56 (70.9\%)	13 (31.7\%)	444 (50.0\%)	
Low	393 (51.2\%)	23 (29.1\%)	28 (68.3\%)	444 (50.0\%)	5.3e‐05*

* Significant.

### 3.6 The cancer‐related hallmark gene sets associated with the lncRNA signature in breast cancer

As presented in Figure S1B, ssGSEA followed by Spearman's correlation analysis was performed, and it was found that the hallmark gene sets significantly associated with the lncRNA signature included mTORC1 signaling (*r* = 0.607, *p *< 0.001), G2M checkpoints (*r* = 0.516, *p *< 0.001), E2F targets (*r* = 0.476, *p *< 0.001), unfold protein response (*r *= 0.430, *p *< 0.001), mitotic spindle (*r* = 0.420, *p *< 0.001), glycolysis (*r* = 0.409, *p *< 0.001), and MYC targets V1 (*r* = 0.408, *p *< 0.001).

**FIGURE 8:**
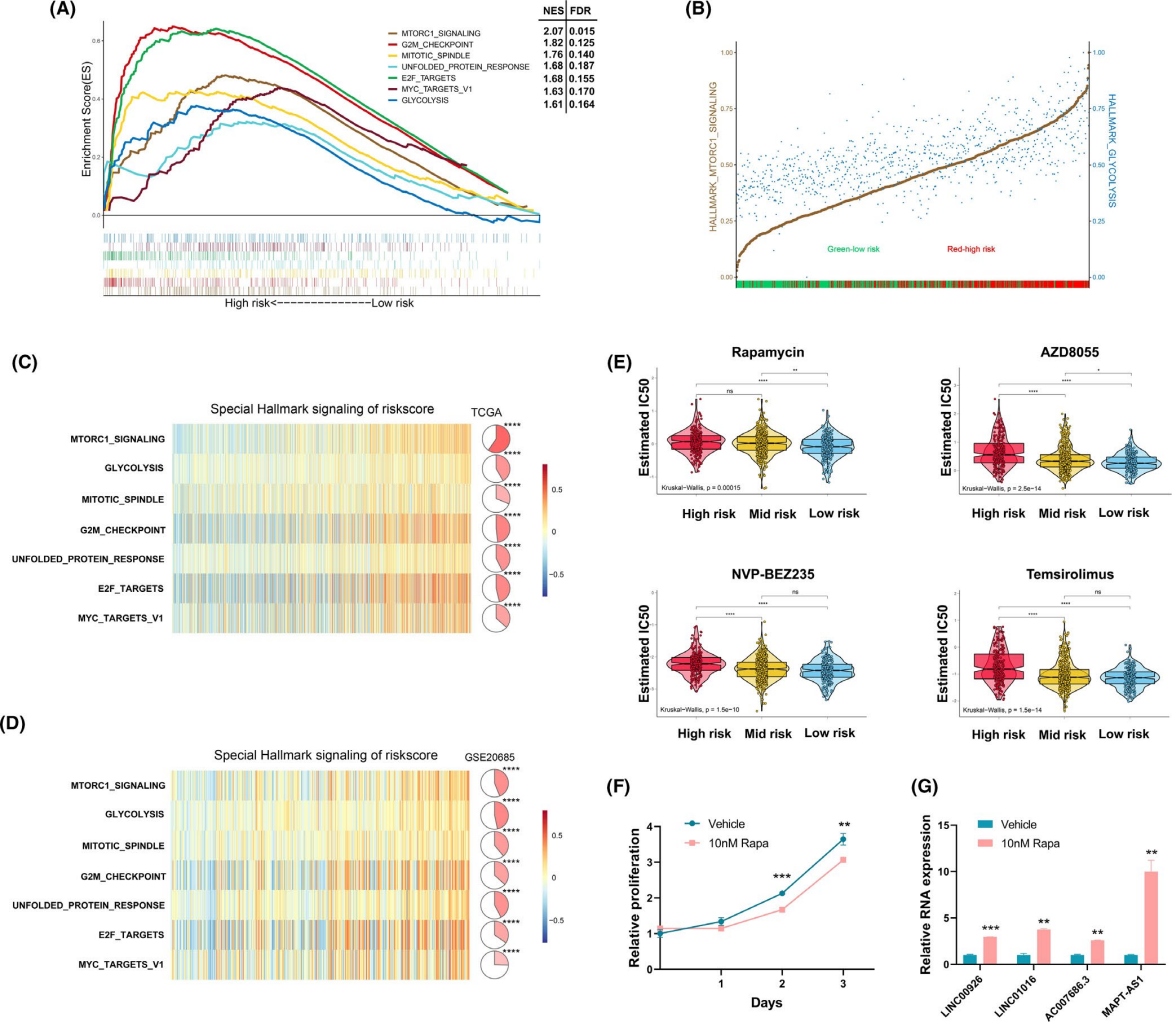
The cancer‐related hallmark gene sets associated with the lncRNA signature in breast cancer. (A) Gene set enrichment analysis. (B) The interplay between the lncRNA signature, mTORC1 signaling, and glycolysis signaling. Brown: the ssGSEA score of mTORC1 signaling; Blue: the ssGSEA score of glycolysis signaling; Red: high‐risk patients; Green: low‐risk patients. The ssGSEA score was scaled to a range between 0 and 1 in the plot. (C, D) Gene set variation analysis. (E) The GDSC drug response data were used to estimate the association between the lncRNA signature and mTOR inhibitors in TCGA breast cancer patients. (F) Proliferation of MDA‐MB‐231 cells treated with vehicle control or 10 nM rapamycin. (G) Signature lncRNAs with significant expression change in response to rapamycin treatment (*p < 0.05, **p < 0.01, ***p < 0.001, ****p < 0.0001)

Based on the median risk score, TCGA‐derived patients were stratified into two subgroups, high‐ and low‐risk groups. GSEA showed that the mTORC1 signaling pathway was most significantly enriched in the high‐risk group (NES = 2.07, FDR = 0.015) (Figure 8A), which suggested that the lncRNA signature may contribute to the regulation of the mTORC1 signaling pathway. Moreover, the interplay between mTORC1 signaling, glycolysis signaling, and the prognostic signature is shown in Figure 8B, which revealed that mTORC1 signaling was significantly positively correlated with glycolysis signaling, and the high‐risk group displayed higher levels of enrichment for mTORC1 and glycolysis signaling compared to the low‐risk group.

To validate and further clarify the association between the lncRNA signature and hub hallmark gene sets, we performed GSVA as described in Figure 8C,D. Accordant with the above results, we observed that several hallmark gene sets related to cell growth were significantly upregulated in the training and validation sets; these gene sets included mTORC1 signaling, G2M checkpoints, E2F targets, unfold protein response, mitotic spindle, glycolysis, and MYC targets V1.

To estimate whether the lncRNA signature can predict clinical response to mTOR inhibitors, we extracted the data of related drugs from the Genomics of Drug Sensitivity in Cancer (GDSC),^28^ including four mTOR inhibitors (Rapamycin, AZD8055, NVP‐BEZ235, and Temsirolimus). Our results indicated that the high‐risk group appeared as a higher IC50 value for mTOR inhibitors than the low‐risk group (Figure 8E). We next treated MDA‐MB‐231 cells with 10 nM rapamycin, an mTORC1 inhibitor. MTT assay showed that rapamycin indeed resulted in cell growth inhibition compared to vehicle‐treated group (Figure 8F). On the other hand, expression levels of LINC00926, LINC01016, AC007686.3, and MAPT‐AS1 also significantly increased in response to rapamycin treatment (Figure 8G), which indicated that the mTORC1 signaling pathway was negatively associated with the expression of signature lncRNAs.

### 3.7 Cell growth‐related signaling significantly activated in the high‐risk group

**FIGURE 9:**
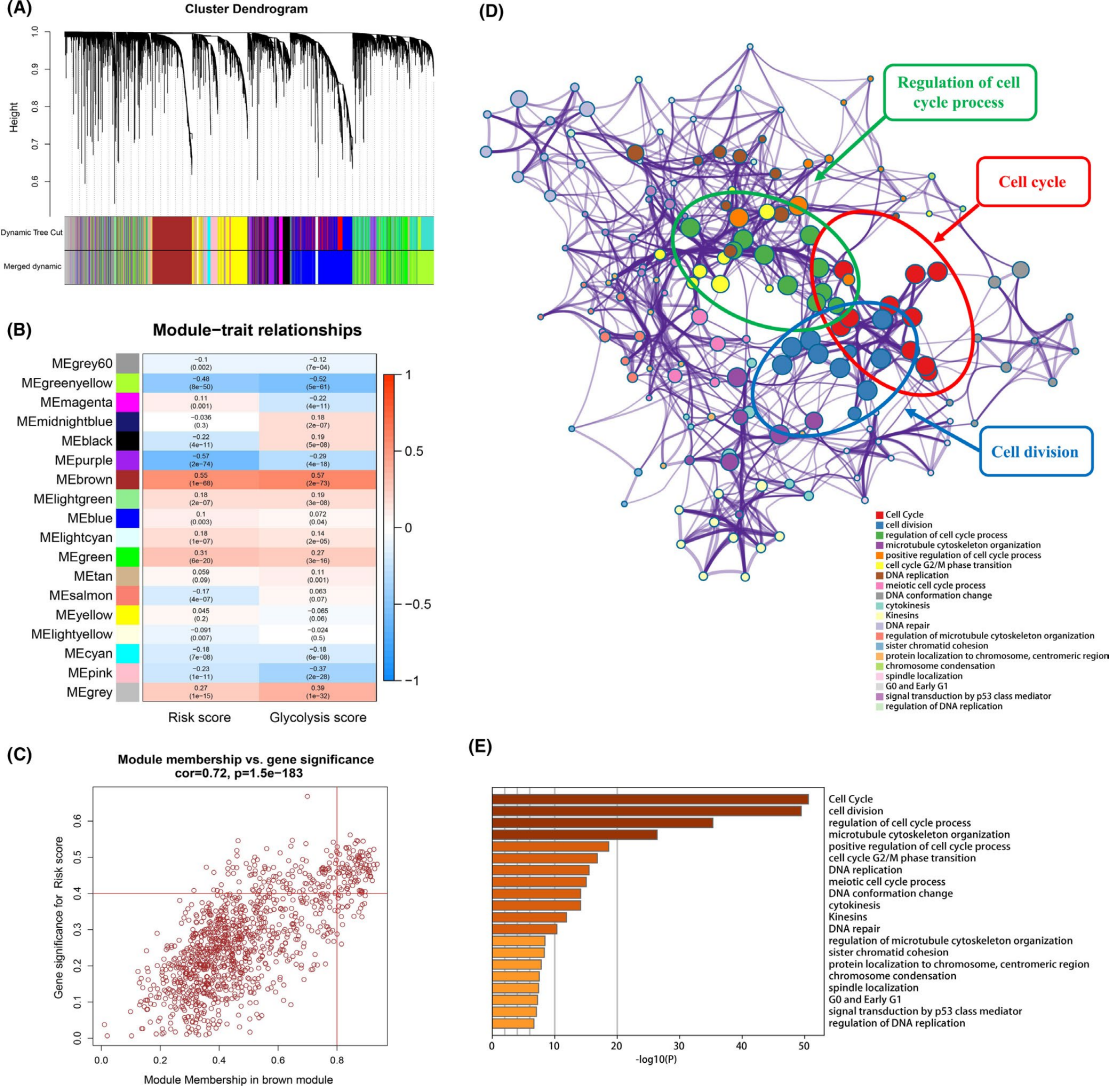
Cell growth‐related signaling significantly activated in the high‐risk group. (A) Clustering dendrogram of mRNAs. The two colored rows represent the original modules and merged modules, respectively. (B) The relationship between modules and traits. (C) A scatter plot of GS for risk scores versus MM for brown module. Red line represents the screening criteria: MM value greater than 0.8 and GS value greater than 0.4. (D, E) Biological process enrichment analysis of 93 hub genes from the brown module

Given that the hallmark gene sets related to cell growth were upregulated in the high‐risk group, we continued to perform WGCNA to identify the biological processes involved in the lncRNA signature. As presented in Figure 9A, the genes enrolled in the training set were clustered into 18 modules using cluster analysis. Subsequently, the brown module was found to be highly associated with the lncRNA signature (Figure 9B). In the brown module, 93 hub genes were selected based on the criteria of MM values greater than 0.8, and a GS value greater than 0.4 (Figure 9C; Table S5). Finally, biological process enrichment analysis of hub genes from the brown module was performed using Metascape. As expected, we found that cell growth‐related signaling, including cell cycle, cell division, and regulation of cell cycle process, were significantly enriched in the high‐risk group (Figure 9D,E). Taken together, the lncRNA signature may be associated with tumor malignant progression and higher mortality risk by promoting tumor cell proliferation.

## 4 DISCUSSION

For decades, great advances have been made in breast cancer treatment; however, several mechanisms associated with breast cancer progression remain elusive.^29^, ^30^, ^31^, ^32^ Reprogrammed energy metabolism is currently identified as an emerging hallmark of cancer cells.^6^ This alteration is characterized by preferential dependence on glycolysis for energy production even in the presence of adequate oxygen and fully functioning mitochondria, namely ‘aerobic glycolysis’ or ‘Warburg effect’.^3^, ^9^, ^33^ Furthermore, previous studies have shown that tumor aerobic glycolysis frequently contributes to poor clinical outcomes in patients with breast cancer.^34^, ^35^, ^36^ Thus, continued investigation of aerobic glycolysis could help to gain insight into the crucial mechanism of breast cancer initiation and progression and develop better prognostic indicators, diagnostic biomarkers, and therapeutic targets for breast cancer patients.

LncRNAs were previously reported to be involved in tumor metabolism reprogramming.^37^, ^38^, ^39^, ^40^, ^41^ In this study, we developed a glycolysis score to further construct a five glycolysis‐related lncRNA signature, which was associated with malignant progression of breast cancer and acted as an independent prognostic factor in breast cancer patients. Subsequent in vitro experiments also supported these findings. Moreover, the lncRNA signature could well distinguish patients with unfavorable prognosis from those with favorable prognosis. Further analyses demonstrated that the lncRNA signature had moderate discrimination for glycolysis status, and the combination of five lncRNAs possessed better predictive efficacy for glycolysis status compared with each lncRNA from the prognostic model. Importantly, a consistent performance was observed in the validation set. In an effort to enhance the predictive efficacy of the lncRNA signature, we further integrated age, AJCC stage, subtype, cancer status, and the lncRNA signature to develop a nomogram which predicts the efficacy for survival and clinical utility, and was validated by calibration curve, C‐index, and DCA, respectively. Lastly, our findings suggest that the nomogram based on the lncRNA signature could contribute to predicting survival probability and help to guide personalized therapeutic strategies for breast cancer patients.

lncRNAs play pivotal roles in metabolism reprogramming of breast cancer by regulating important cancer‐related pathways.^42^, ^43^, ^44^ GSEA of hallmark gene sets was performed and identified that mTORC1 signaling was significantly enriched in the high‐risk group. In addition, we observed that mTORC1 signaling was positively correlated with glycolysis signaling. According to previous reports, the mTOR signaling pathway could integrate both intracellular and extracellular signals and function as a central pathway in tumor initiation and progression.^45^, ^46^, ^47^ Accumulating evidence also demonstrated that the mTORC1 signaling pathway may act as a mediator of aerobic glycolysis to promote cell proliferation.^45^, ^48^, ^49^ Subsequent GSVA further identified that the risk score was positively correlated with the mTORC1 signaling pathway as well as other hallmark gene sets associated with cell growth. Notably, the results were mutually validated in two independent datasets.

To further investigate the biological processes related to the lncRNA signature, we applied WGCNA and identified that the brown module was highly associated with the risk score and glycolysis score. Furthermore, Metascape was conducted and demonstrated that hub genes sourced from the brown module were significantly enriched in cell growth‐related signaling, which could promote tumor cell proliferation and contribute to a higher mortality risk in breast cancer patients.

When glycolysis inhibitors are employed, mTORC1 could be involved in metabolism reprogramming to escape from glycolytic dependency.^50^ Currently, mTOR inhibitors have been used in clinical practice. Therefore, we tried to estimate the association between the lncRNA signature and drug response via the GDSC drug response data and in vitro experiments. Finally, our data suggested that the lncRNA signature can serve as a promising indictor for measuring response to mTOR inhibitors in breast cancer patients. Moreover, previous studies have shown that tumor cell proliferation could be inhibited by co‐targeting glycolytic enzyme and mTORC1 signaling.^50^ Given that the lncRNA signature had a significantly positive association with mTORC1 and glycolysis signaling, it may help in developing novel therapeutic strategies for combination therapy, and achieving desirable clinical benefits for breast cancer patients.

Our data presented here provided a basis for further exploration of metabolic strategies in breast cancer prognosis and treatment. However, this study was mainly based on the publicly available datasets. Because the public sample size is limited, further exploring these findings will be a crucial direction for our future work.

In conclusion, we identified five glycolysis‐related lncRNAs to construct an lncRNA signature on the basis of the glycolysis score, which could predict the survival probability and glycolysis status. Moreover, hallmark gene sets associated with cell growth were significantly activated in the high‐risk breast cancer patient subgroup. Overall, the lncRNA signature could function as a robust prognostic indicator and help to develop novel therapeutic strategies for breast cancer patients.

## CONFLICT OF INTEREST

No potential conflict of interest was reported by the authors.

## ETHICAL APPROVAL

All analyses in this study were based on the publicly available database. No ethical approval and patient consent are required.

## Supporting information

Figure S1

Table S1

Table S2

Table S3

Table S4

Table S5

## Data Availability

The datasets analyzed in this study were sourced from the TCGA (http://cancergenome.nih.gov/), METABRIC (https://www.mbcproject.org/), GEO (https://www. ncbi.nlm.nih.gov/geo/), and GDSC (https://www.cancerrxgene.org/).
