# Supplementary material for: Integrated analysis identifies a novel lncRNA prognostic signature associated with aerobic glycolysis and hub pathways in breast cancer
Source: Cancer Med. 2021 Sep 27;10(21):7877–92. doi: 10.1002/cam4.4291 (PMC8559482; doi:10.1002/cam4.4291)
Supplement: Supplementary file 1 — Figure S1 [file CAM4-10-7877-s004.docx]

**Supplementary Figure S1**


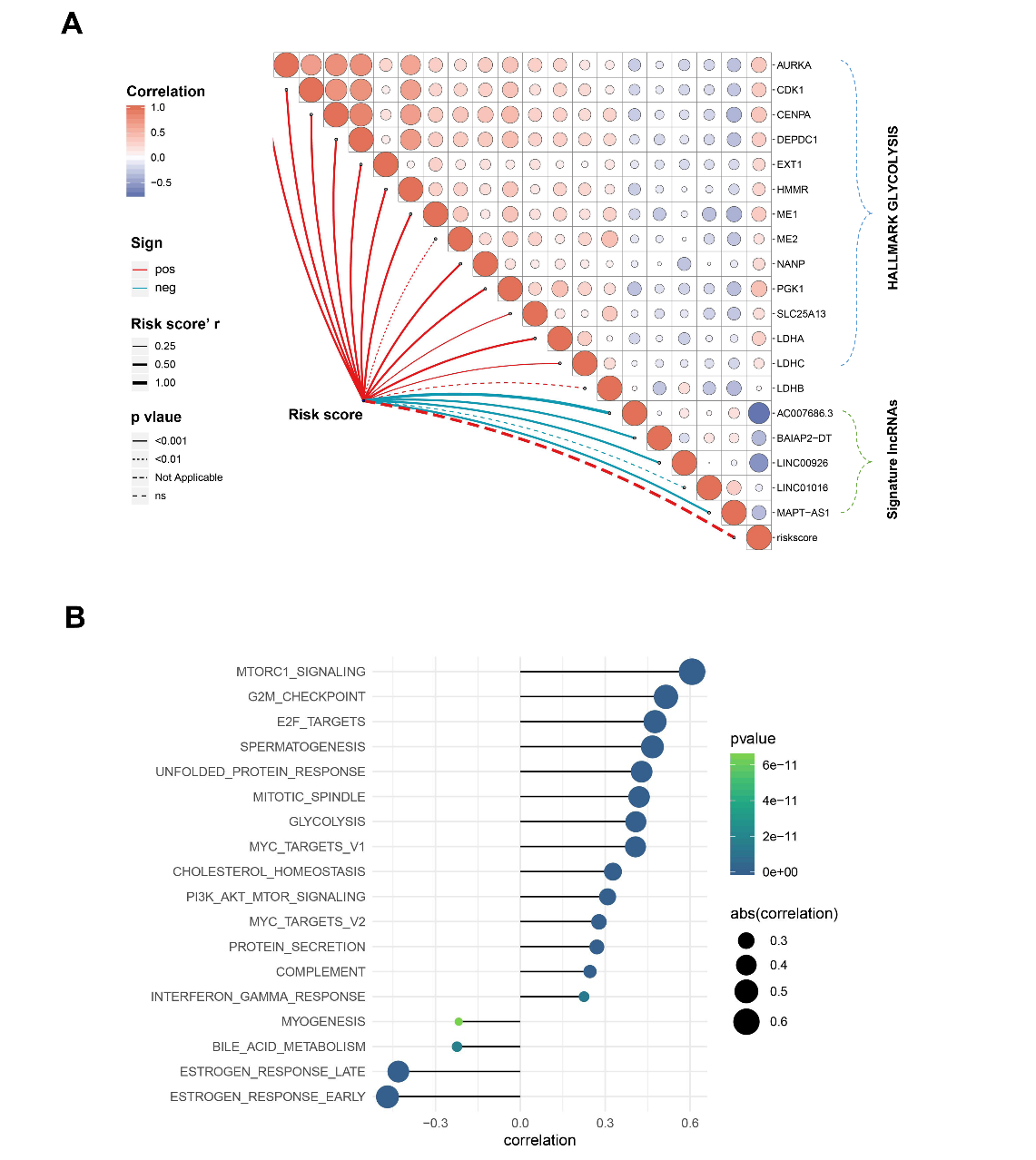


**Figure S1** A, The relationship among aerobic glycolysis-related factors, lncRNA signature, and each lncRNA in the validation set. B, ssGSEA followed by Spearman’s correlation analysis were performed to identify the hallmark gene sets significantly associated with the lncRNA signature.
