# Supplementary material for: Integrated analysis identifies a novel lncRNA prognostic signature associated with aerobic glycolysis and hub pathways in breast cancer
Source: Cancer Med. 2021 Sep 27;10(21):7877–92. doi: 10.1002/cam4.4291 (PMC8559482; doi:10.1002/cam4.4291)
Supplement: Supplementary file 3 — Table S2 [file CAM4-10-7877-s003.docx]

**Supplementary Table S2. Primers used for real-time PCR**

| **Gene** | **Forward （5’-3’）** | **Reverse （5’-3’）** |
| --- | --- | --- |
| LINC00926 | TTAGTAGGGACGAGGTTTCA | ATTGGCTTAGTTCTTCTTGG |
| LINC01016 | AAGGCATGAGACTCAATCA | AATCAACTAAGAGCCAGGTA |
| AC007686.3 -201 | TCAGAGTGCGCTACCCTTCC | AAACTGCGGCTGTGCTTTAC |
| MAPT-AS1 | GGAGCTTGGCAGTCCAGGTT | CAGAGACACACAGGGAGAATGC |
| BAIAP2-DT | CTCCACCAAGGATGTTCCCC | AAATGCGTCGGGACAAGAGT |
| Actin | CATGTACGTTGCTATCCAG | CTCCTTAATGTCACGCACG |
